# Prenatal SAMe Treatment Induces Changes in Brain Monoamines and in the Expression of Genes Related to Monoamine Metabolism in a Mouse Model of Social Hierarchy and Depression, Probably via an Epigenetic Mechanism

**DOI:** 10.3390/ijms231911898

**Published:** 2022-10-07

**Authors:** Maria Becker, Karin Abaev, Elena Shmerkin, Liza Weinstein-Fudim, Albert Pinhasov, Asher Ornoy

**Affiliations:** 1 Department of Morphological Sciences and Teratology, Adelson School of Medicine, Ariel University, Ariel 4076414, Israel; 2 Department Molecular Biology, Adelson School of Medicine, Ariel University, Ariel 4076414, Israel; 3 Department of Medical Neurobiology, Hebrew University Hadassah Medical School, Jerusalem 9112102, Israel

**Keywords:** S-Adenosyl-methionine, depression, brain monoamine metabolism

## Abstract

Reduction in the levels of monoamines, such as serotonin and dopamine in the brain, were reported in patients and animals with depression. SAMe, a universal methyl donor and an epigenetic modulator, is successfully used as an adjunct treatment of depression. We previously found that prenatal treatment with SAMe of Submissive (Sub) mice that serve as a model for depression alleviated many of the behavioral depressive symptoms. In the present study, we treated pregnant Sub mice with 20 mg/kg of SAMe on days 12–15 of gestation and studied the levels of monoamines and the expression of genes related to monoamines metabolism in their prefrontal cortex (PFC) at the age of 3 months. The data were compared to normal saline-treated Sub mice that exhibit depressive-like symptoms. SAMe increased the levels of serotonin in the PFC of female Sub mice but not in males. The levels of 5-HIAA were not changed. SAMe increased the levels of dopamine and of DOPAC in males and females but increased the levels of HVA only in females. The levels of norepinephrine and its metabolite MHPG were unchanged. SAMe treatment changed the expression of several genes involved in the metabolism of these monoamines, also in a sex-related manner. The increase in several monoamines induced by SAMe in the PFC may explain the alleviation of depressive-like symptoms. Moreover, these changes in gene expression more than 3 months after treatment probably reflect the beneficial effects of SAMe as an epigenetic modulator in the treatment of depression.

## 1. Introduction

Different studies have suggested that imbalances in the levels of biogenic amines, such as dopamine (DA) and serotonin (5-HT or 5-hydroxytryptamine) are involved in the etiology of a variety of psychiatric disorders such as schizophrenia, attention-deficit/hyperactivity disorder, and depression [1,2]. The monoamine theory of depression postulated that aberrant function of brain monoamine neurotransmitters 5-hydroxytryptamine (5-HT or serotonin), dopamine (DA), and norepinephrine (NE) leads to perturbation in certain brain circuits related to emotional control [2]. Functional and molecular alterations in the prefrontal cortex (PFC) and hippocampus (HPC) and monoamine synaptic imbalance have been associated with Major Depressive Disorder (MDD) [2,3]. In MDD, the CSF and jugular vein plasma levels of the dopamine (DA), DA metabolite, 3,4-dihydroxyphenylacetic acid (DOPAC) and homovanillic acid (HVA) are abnormally decreased, consistent with decreased DA turnover defined as the ratio of HVA to DA [4,5]. Reduced CSF concentrations of monoamine metabolites, 5-hydroxyindoleacetic acid (5-HIAA), HVA, and 3-methoxy-4-hydroxyphenylglycol (MHPG) were detected in MDD patients [6]. Monoamines metabolism and turnover represent ongoing equilibrium between synthesis, storage and catabolic degradation that reflects the catecholamine release in response to increased nerve impulse activity [7].

S-Adenosyl-methionine (SAMe, also known as AdoMet), is an important methyl donor synthetized by all living organisms (reviewed in [8]). Long-term epigenetic changes occur during prenatal or early postnatal developmental phases when epigenetic reprogramming occurs and may underlie the predisposition to development of depression. SAMe also regulates synthesis and function of monoamine neurotransmitters, as serotonin, dopamine and noradrenaline [9]. Administration of substrates such as L-dihydroxyphenylalanine, that facilitates dopamine synthesis, led to a prominent decrease in SAMe concentrations in the brain of rats [10], in whole blood [11] and CSF in humans [11]. Moreover, SAMe concentrations were also reduced in rat brain tissue following treatment with monoamine reuptake inhibitors [12]. SAMe was also suggested to be a potent epigenetic drug [8,13].

In the last decade, there has been a considerable focus on the role of epigenetics as a possible bridge between genes and experience in the pathophysiology of depression. Great emphasis is put on the epigenetic changes triggered by exposure to various extrinsic factors (such as environmental pollution, toxins, viral infections, maternal nutrition and metabolism) in utero and at early-life that lead later in life to adult-onset diseases [14,15]. Epigenetic changes triggered by various extrinsic factors during prenatal and early postnatal life can affect the normal physiological and metabolic functions at adulthood [14,16]. Epigenetic mechanisms may, in part, mediate the influence of environmental stress and interact with genetic liability for MDD over the lifespan [17,18].

Submissive (Sub) mice, originating from the Sabra strain, serve as an animal model of depression demonstrating depressive-like behavior and are susceptible to stressful stimuli [19,20]. The evaluation of the depression-like behavior of these Sub male and female mice demonstrated that prenatal treatment with SAMe in Submissive mice did not affect the early neurodevelopmental milestones in males or females but improved depression-like behavior, especially social impairment, when compared to saline treated mice [21]. Some of these improvements were gender related. The impaired sociability of Sub mice was significantly improved in both genders following SAMe treatment. SAMe’s beneficial effect in the classic Porsolt behavioral despair test was moderate, being observed only during the last 2 min test time interval and only in females.

Sub mice have impaired locomotion that apparently reflects the absence of motivation [22,23], which is mainly regulated by the dopaminergic system and less by the serotonergic system. Behavioral phenotype is associated in sub mice with emotionality and social hierarchy and is reflected by the altered monoamines content in various brain areas [24]. Decreased levels of 5-HT in the brainstem, reduced levels of norepinephrine in the prefrontal cortex and hippocampus, and elevated levels of dopamine in the prefrontal cortex, hippocampus, striatum and brainstem were also measured in Sub mice [24].

We estimated the effects of prenatal SAMe treatment on offspring’s monoamine neurotransmitters turnover in the PFC by calculating the ratio between local neurotransmitter concentrations and their degradation products that reflect the neuronal activity and monoamines release. We investigated whether monoamine concentrations and their degradation metabolites were altered in the PFC and whether such alterations were associated with changes in the expression of genes coding for enzymes involved in their catabolic degradation and the expression levels of genes coding for serotonin receptor 5-HT_2A_ (5-HT_2A_R) and dopamine receptors D_1_ (D_1_R) and D_2_ (D_2_R). We believe that epigenetic changes related to monoamines may be responsible at least in part for the depressive–like behavioral trait in the Sub mice.

## 2. Results

### 2.1. SAMe Effect on Serotonin Metabolism in the PFC

**Figure 1:**
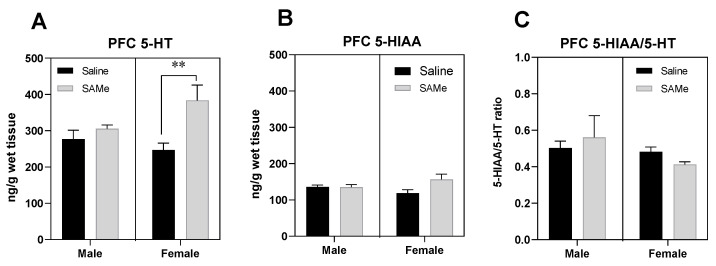
SAMe effects on Serotonin turnover in the PFC. SAMe treatment significantly elevated the levels of serotonin in a gender related manner. SAMe treatment effect was significant only in female Sub mice (**A**). (**B**) The levels of 5-HIAA were not changed by SAMe in both males and females. (**C**) the 5-HIAA/5-HT ratio remained unchanged following SAMe treatment in both male and female. The data are presented as mean ± SEM. Levels of 5-HT and 5-HIAA are presented in ng/g brain tissue, n ≥ 6 for each group. ** *p* < 0.01 compared to control mice of the same gender.

The activity of 5-hydroxytryptaminergic neurons was estimated by measurements of the brain tissue levels of 5-HT and its primary end-product 5-HIAA, and by the ratio of 5-HIAA to 5-HT in the PFC of Sub mice offspring prenatally treated with SAMe or Saline (Figure 1). Moreover, 5-HIAA is the primary metabolic product of enzymatic degradation of 5-HT by monoamine oxidase A (MAO-A); and ratio of 5-HIAA to 5-HT provide an indication of the serotonin turnover rate in the brain. The 5-HIAA-to-5-HT ratio served as an estimated index of the changes in the rate of release of 5-HT into the synapse.

SAMe treatment elevated the levels of 5-HT in the PFC compared to control mice only in female offspring of Sub mice (Figure 1A). Two-way ANOVA revealed significant effect of SAMe treatment (F (1, 6) = 9.450, *p* < 0.02), but no effect of gender. Paired *t*-test analysis of individual groups indicated the significantly higher levels of 5-HT in SAMe treated female PFC (*p* < 0.03) in comparison to saline treated group. (Figure 1A).

Two-way ANOVA analysis of 5-HIAA concentrations demonstrated no effects of SAMe treatment (F (1, 6) = 1.807, *p* ≥ 0.05) and of gender. SAMe treatment did not change the levels of 5-HIAA compared to controls (Figure 1B). The 5-HIAA/5-HT ratio in the PFC was unchanged too following SAMe treatment (Figure 1C).

### 2.2. SAMe Effect on Dopamine, Dopamine Metabolites and of Metabolites/Dopamine Ratio in the PFC

**Figure 2:**
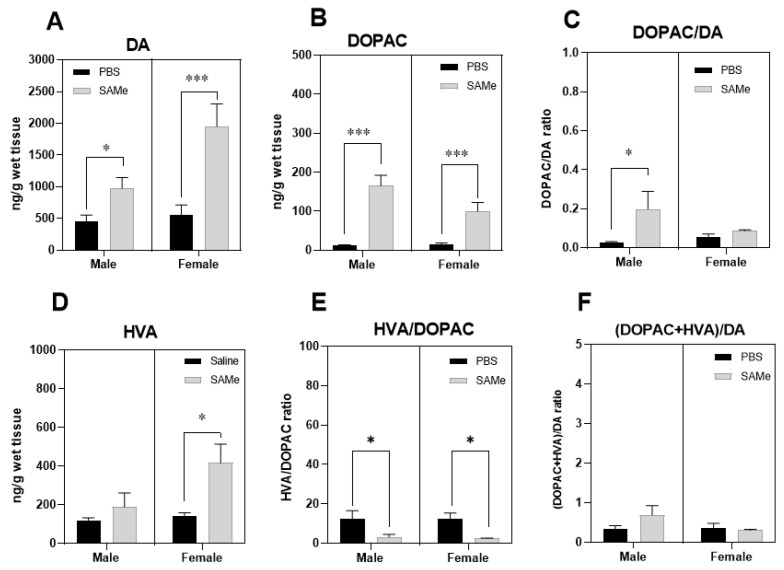
SAMe effects on dopamine metabolism in the PFC. SAMe treatment significantly elevated the level of dopamine (**A**), and of DA catabolic product-DOPAC (**B**) in the PFC of Sub male and female mice and lead to prominent increase in DOPAC/DA index only in male PFC (**C**). SAMe treatment also induced higher levels of HVA (**D**), the end product of DA degradation only in females and decreased HVA/DOPAC index in both genders (**E**). However, the overall turnover of DA was unchanged (**F**). The data are presented as mean ± SEM. Levels of DA, DOPAC and HVA are presented in ng/g tissue, n ≥ 6 for each group. *** *p* < 0.001; * *p* < 0.05 compared to control mice of the same gender.

Two-way ANOVA analysis of dopamine level in the PFC of sub mice revealed significant effect of SAMe treatment (F (1, 6) = 6.3, *p* < 0.04) and of sex (F(1, 6) = 14.3, *p* < 0.009). Paired *t*-test comparison defined near two-fold elevation of DA concentrations in SAMe treated male offspring (*p* < 0.026) and a strong, 3.5-fold increase in female offspring (*p* < 0.003) (Figure 2A) compared to controls.

For estimation of the catabolic rate of DA degradation we initially measured DOPAC a first step metabolic product of enzymatic degradation of DA initially by MAO, followed by aldehyde dehydrogenase (ALDH), and then HVA degraded from DOPAC by COMT during the second step of degradation. We estimated several catabolic indexes of DA turnover by calculation of DOPAC to DA ratio, HVA to DOPAC ratio and finally the DOPAC + HVA to DA ratio that served as an overall estimated index of the dopaminergic activity in the PFC.

SAMe significantly raised DOPAC level in the offspring of Sub mice compared to controls (F(1, 6) = 42.3, *p* < 0006 of treatment effect). Multiple *t*-test of paired comparison demonstrated higher concentrations of DOPAC in either male (*p* < 0.0006) or female (*p* < 0.012) offspring (Figure 2B). However, DOPAC to DA ratio was statistically higher only in male Sub mice PFC, but not in female when compared to saline treated mice (Figure 2C).

Two-way ANOVA analysis of HVA to DOPAC ratio revealed the effect of SAMe treatment (F(1, 6.) = 24.87, *p* < 0.0025. Figure 2E), but no differences in the final DOPAC + HVA to DA ratio (Figure 2F). Multiple *t*-test paired comparisons showed significant decrease in the HVA to DOPAC ratio in either male (*p* < 0.049) or female (*p* < 0.013) offspring of Sub mice after SAMe treatment.

Two-way ANOVA showed significant SAMe treatment effect on the concentration of HVA metabolite in the offspring of Sub mice (F(1, 6) = 7; *p* < 0.04). Multiple *t*-test paired comparisons showed significant difference in HVA levels in female offspring (*p* < 0.012) after SAMe treatment (Figure 2D) but not in males when compared to controls. HVA/DOPAC ratio was decreased in both genders (Figure 2E) and the DOPAC + HVA/DA ratio was unchanged (Figure 2F).

### 2.3. SAMe Effect on Norepinephrine Metabolism in the PFC

The activity of norepinephrine neurons was estimated by measurements of the brain tissue levels of NE, its major metabolite MHPG and by the ratio of MHPG to NE. MHPG is a major metabolic product of enzymatic degradation of NE involving COMT, MAO-A and an aldehyde dehydrogenase enzyme (reviewed in [7]). Its levels are therefore used as an indication of the NE degradation in the brain. Analysis of NE and MHPG content, and MHPG to NE ratio demonstrated no effect of SAMe treatment on NE activity in comparison to control mice.

### 2.4. SAMe Effects on Tph2, Mao-a, Mao-b and Compt Gene Expression in the PFC

We measured the expression level of *Tph2* gene coding the enzyme that participates in conversion of tryptophan into serotonin within the nerve cell body. In addition, we measured the expression levels of two monoamine oxidases genes, A and B (*Mao-a* and *Mao-b*) that catabolize serotonin into 5-HIAA and are also involved in the dopamine degradation pathway. We evaluated the expression level of *Comt* gene coding the enzyme COMT that is involved in the dopamine degradation pathway and, like MAO enzymes, is involved in the formation of DOPAC and HVA as the main end-metabolites.

**Figure 3:**
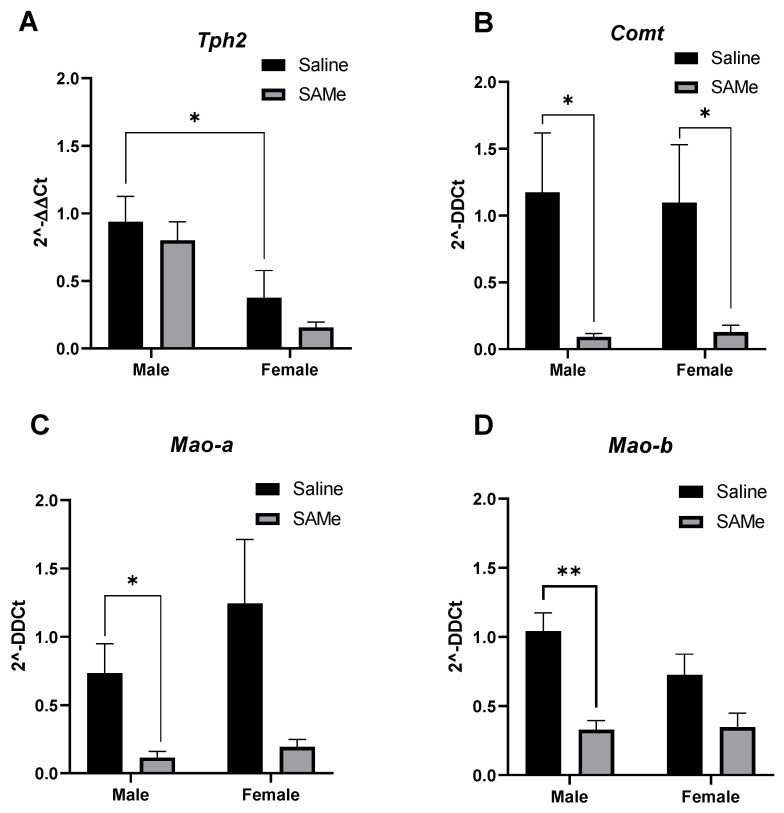
*Tph2, Mao-a*, *Mao-b* and *Comt* genes expression in the PFC. SAMe treatment significantly decreased the expression of *Mao-a* (**C**) and *Mao-b* (**D**) genes only in male Sub mice and decreased the level of *Comt* (**B**) in both genders. No effect of SAMe treatment was measured on *Tph2* (**A**) gene expression in either males or females. The data are presented as means ± SEM, * *p* < 0.05 or ** *p* < 0.002.

Two-way ANOVA analysis revealed significant sex difference in the expression of *Tph2* gene in Sub mice (F (1, 6) =10.16; *p* < 0.009) but no effect of treatment (Figure 3A).

SAMe significantly decreased the expression of *Mao-a* gene as observed by two-way ANOVA analysis (F (1, 5) =2.74, *p* < 0.016). Multiple paired *t*-test demonstrated that *Mao-a* gene expression was lower (*p* = 0.023) in male Sub mice offspring treated with SAMe. In females we observed only a trend (*p* = 0.09) that did not reach statistical significance in comparison to controls (Figure 3B).

### 2.5. SAMe Treatment Effects on Htr2a, Sert, D_1_R and D_2_R Gene Expression in the PFC

**Figure 4:**
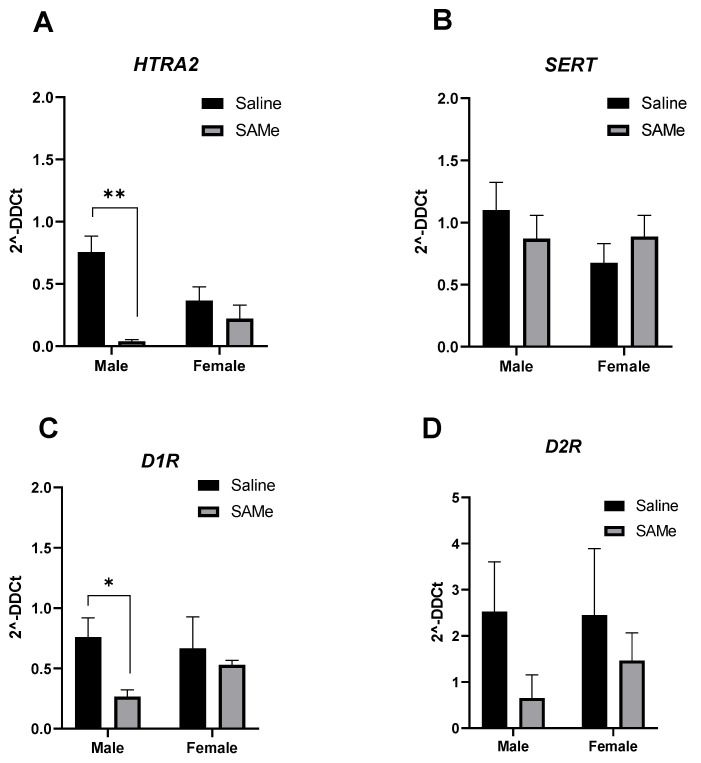
*Htr2a, Sert,* D_1_R and D_2_R genes expression. SAMe treatment significantly decreased the expression of *Htr2a* (**A**) and *D_1_R* (**C**) genes only in male Sub mice. No effect of SAMe treatment was measured on *Sert* (**B**) and *D_2_R* (**D**) gene expression in either males or females. The data are presented as means ± SEM, * *p* < 0.05 or ** *p* < 0.002.

Two-way ANOVA analysis of expression level of *Htr2a* receptor gene determined the effect of SAMe treatment (F (1, 6) = 10.76; *p* = 0.022). Multiple paired *t*-test demonstrated prominent reduction in the expression of *Htra2* gene (*p* = 0.0002) only in males in comparison to controls (Figure 4C).

Multiple paired *t*-test defined the decrease in D_1_R gene expression only in males treated with SAMe in comparison to controls (*p* = 0.62). No significant differences were measured for the expression of *Sert* and D_2_R genes among genders and experimental groups.

Table 1 is a summary of the SAMe—induced changes in the monoamine levels and in the expression of related genes. Some of the changes are similar in both sexes and some are different between males and females (Table 1).

## 3. Discussion

The pathophysiology and etiology of depression remains unknown despite numerous studies that have been carried out. In patients with major depressive disorder either serotonin or dopamine levels or both have been found to be reduced [25,26].

Using a HPLC-ECD methodology we assessed serotonergic compounds (5-HT and 5-HIAA), dopaminergic (DA, DOPAC and HVA) and noradrenergic (NA and MHPG) in the PFC of Sub offspring prenatally treated with SAMe in comparison to the levels in control saline treated Sub mice. The obtained data were also compared to assessed changes of gene expression of particular genes involved in the anabolic and catabolic processes of these monoamine systems, such as *Tph2*, *Mao* (*Mao-a and Mao-b*), *Comt* and certain serotonin and dopamine receptors genes, *Ht2a*, D_1_R and *D_2_R* and serotonin re-uptake transporter gene, *Sert*. Taken together, SAMe treatment seems to regulate monoamine concentrations in the PFC probably via epigenetic mechanisms, explaining the beneficial effects of SAMe as a treatment modality in patients with depression. This also explains the improvement in several behavioral parameters of depression in our Sub mice following prenatal SAMe treatment, as published by us previously [20].

### 3.1. SAMe Effects on the Serotonergic Metabolism

We found increased levels of 5-HT in Sub females prenatally treated with SAMe but not in males, while the levels of 5-HIAA or 5-HT to 5-HIAA ratio were unchanged.

These results may indicate two possible processes in relation to elevated 5-HT; the first that 5-HT synthesis was higher in female Sub mice following SAMe treatment or, that serotonin degradation processes were slowed down. It was shown that acute paroxetine treatment (i.p, 3 mg/kg) which is known to decrease serotonin degradation, elevated 5-HT level in the PFC of Sub mice and decreased the level of 5-HIAA and the ratio of 5-HT to 5-HIAA [24]. Hence, it is reasonable to assume that SAMe has a similar effect to that of Paroxetine.

Serotonin is a monoamine molecule that is synthesized from tryptophan by TPH_2_ enzyme in the various cell groups in the medulla, pons and mesencephalon. Generally, tryptophan hydroxylase 2 (*Tph2*) gene specifically expressed in the brain is inducing TPH_2_, an important enzyme responsible for serotonin availability in neuronal cells [27]. Serotonin in the forebrain is synthesized by 5-HT dorsal raphe nuclei neurons that project to the cortex and striatum [28]. The activity of serotonergic neurons may be assessed by the concentration of 5-Hydroxyindoleacetic acid (5-HIAA) a primary metabolite of 5-HT [6,28,29,30]. Decreased serotonergic activity was found in brainstem and prefrontal cortex of depressed people that committed suicide, where the ratio of 5-HIAA to 5-HT was lower compared with controls [29]. Patients with MDD have higher 5-HIAA in jugular venous blood apparently reflecting higher brain 5-HT neurotransmission and turnover [6,30,31]. In the CSF of MDD patients, the concentrations of 5-HIAA were also reduced [6]. However, these changes may have been associated with antidepressant drugs treatment [32,33].

Many studies suggested that lower serotonin activity corresponded with atrophy of certain PFC regions in patients with depression [34,35]. Studies of acute tryptophan depletion, that leads to 5-HT reduced production and to decreased serotonergic activity have shown that serotonin may underlie the etiology and possibly sex dimorphism of anxiety and major depression [36,37]. Moreover, the depressive symptoms were significantly greater in women during tryptophan depletion [36]. In another study, tryptophan depletion did not lead to depressive symptoms in healthy participants without depression but induced depressive symptomatology in depressed patients in remission free of medications [38]. The role of serotonin and their metabolites in the etiology of depression was recently revised by Moncrieff et al. [39]. The authors estimated that there is ”no consistent support for the hypothesis that depression is caused by lowered serotonin activity or concentrations” [39].

Sub mice have impaired locomotion that apparently reflects the absence of motivation [22,23]. Previously, we demonstrated increase in locomotion and, possibly, increased motivation in Sub mice prenatally treated with SAMe [21]. The impaired sociability of Sub mice was also significantly improved in both genders following SAMe treatment [21]. Locomotion is mainly regulated by the dopaminergic system and less by the serotonergic system. In addition, SAMe’s beneficial effects in classic Porsolt behavioral despair test were moderate, being observed only during the last 2 min of the test time interval and only in females. Moreover, SAMe treatment did not change anxiety-like behavior referred to emotionality, that is also related to the serotonergic system [20].

Our findings demonstrated that the elevated level of 5-HT induced by SAMe in females, were not associated with changes in the expression levels of *Tph2* gene. However, *Tph2* gene expression shows sex dimorphism in Sub mice, with higher expression in male than in female PFC. In transgenic mice models generated to study the *Tph2* gene polymorphism [40], the depletion or reduced activity of TPH_2_ enzyme did not affect emotional behavior, and only slightly reduced brain serotonin levels. In addition, serotonin metabolite 5-HIAA was dramatically decreased in mice with reduced TPH_2_ activity, possibly by compensating mechanisms to maintain serotonin level in these mice. In *Tph2* null mutant mice (*Tph2*−/−) the deficiency in 5-HT synthesis facilitated panic-like escape behavior as demonstrated by foot shock induced prolonged freezing [40].

SAMe-induced elevated level of 5-HIAA was negatively associated with the decreased levels of gene expression coding the principal enzyme MAO-A that catabolizes 5-HT into 5-HIAA. SAMe downregulated the expression of *Mao-a* gene in male PFC, with slightly, but non –significant, *Mao-a* gene downregulation in female PFC.

We have no explanation why female mice have increased serotonin level in their PFC following SAMe treatment, while the level of T*ph2* gene expression remained unchanged; Neither is there an explanation how the downregulation of *Mao-a* gene could reflect the state of increased 5-HT catabolism. The reduction in gene expression may lead to slow serotonin degradation and accumulation of serotonin in the PFC. Additional studies should be performed in order to clarify these issues.

### 3.2. SAMe Effects on Dopaminergic Metabolism

We found increased levels of DA and their metabolite DOPAC in both sexes with increased HVA only in females. These results indicated that DA release and possibly its degradation by MAO enzyme was higher in Sub mice following SAMe treatment. However, degradation processes mediated by COMT enzyme resulted in higher HVA metabolite production only in female PFC, indicating gender-dependent effect of treatment. DOPAC/DA ratio was higher in male Sub mice and HVA/DOPAC ratio was higher in both male and female mice. These findings indicated higher DA metabolism rate in the Sub mice offspring treated by SAMe.

Dopamine is synthesized from l-tyrosine and is mainly produced in neuronal bodies in the midbrain, substantia nigra (SN) and in ventral tegmental area (VTA). The SN axons primarily projects to the striatum (nigrostriatal pathway), whereas the VTA axons project to the limbic (mesolimbic pathway) and cortical areas (mesocortical pathway) [28,41]. DA is involved in regulating motor activity, emotions, motivation, reward and has been associated with cognition [41].

It was previously found that Sub mice have reduced locomotion which may reflect the absence of motivation [22,23]; processes that are under dopaminergic system regulation. Therefore, the current findings of increased dopamine level and their main metabolites DOPAC and HVA induced by SAMe, suggested increased dopaminergic system activity in sub mice brain that reflect the increased sociability index and increased locomotion in the open field test we previously found [20]. In the study of Murlanova et al., [24] acute treatment with paroxetine lead to decreased levels of dopamine in the brain areas of Sub mice, but no alterations in the levels of dopamine metabolites, DOPAC and HVA.

Elevated levels of dopamine and their metabolite DOPAC by SAMe in either male and female offspring was associated with decreased levels of *Mao-a* and *Mao-b* gene expression. However, the decrease in *Mao-a* and *Mao-b* genes was significant only in males while females demonstrated only a trend for reduction of gene expression. It was shown that *Mao-a* gene DNA methylation levels in human peripheral tissue predict activity levels of the enzyme in the brain [42]. Depression in females was associated with hypomethylation in the first exon region of the *Mao-a* gene [43].

Increased levels of HVA, the last metabolite of two dopamine degradation pathways, were only observed in sub female offspring treated by SAMe. HVA is formed via methylation of DOPAC by COMT. It is therefore expected that the expression of *Comt* gene will be increased by SAMe while we found a decrease in its expression. In *Comt* deficient mice, the degradation of dopamine was two-fold slower in the prefrontal cortex compared to wild-type mice [44]. We have no explanation for our findings.

### 3.3. SAMe Effect on Gene Expression Are Possibly via Epigenetic Modulation

Possible involvement of alterations in gene expression in depression and its treatment have been reported by several investigators. Downregulation and methylation of a CpG site in the promoter region of *Tph2* gene were observed in MDD patients who had attempted suicide [45]. It was suggested that *Tph2* might be associated with a lower risk of female MDD [46] or may have a gender dependent effect on susceptibility to MDD. The authors investigated the association between two genetic variants of the *Tph2* gene and MDD in a Chinese Han population [27,47]. TPH_2_ enzyme may also be involved in the regulatory mechanism of SSRIs in the alleviation of depressive symptoms. The long-term administration of SSRI antidepressant fluoxetine was accompanied by up-regulation of *Tph2* mRNA expression [48]. We found the reduction in gene expression of 5-HT_2A_ receptor in male PFC indicated the sex-dependent effect of SAMe treatment. Altered density of 5-HT_2A_R in the brain of depressed subjects was reported by several investigators [49,50]. The density of 5-HT_2A_R was reduced by antidepressant treatment in postmortem prefrontal cortex in patients with MDD. In rats, chronic treatment with citalopram or mirtazapine resulted in decreased mRNA expression and 5-HT_2A_R density [49]. It was indeed postulated that the downregulation of 5-HT_2A_R might be a central mechanism for SSRI antidepressant effect.

## 4. Materials and Methods

**Animals:** All animal experiments were approved by the Institutional Animal Care and Use Committee of Ariel University, Israel and institutional guidelines for the proper and humane use of animals in research were followed (approval # IL-188-12-19). The populations of dominant (Dom) and submissive (Sub) mice were raised from the outbred Sabra strain (Envigo, Jerusalem, Israel), selectively bred for 48 generations based on the food competition Dominance Submissive Relationship (DSR) paradigm [20,51,52].

Submissive female mice were mated with males of the same behavioral phenotype and monitored at early morning for the presence of vaginal plug, which is considered as a gestational day (GD) 0, and the males were removed.

**Figure 5:**
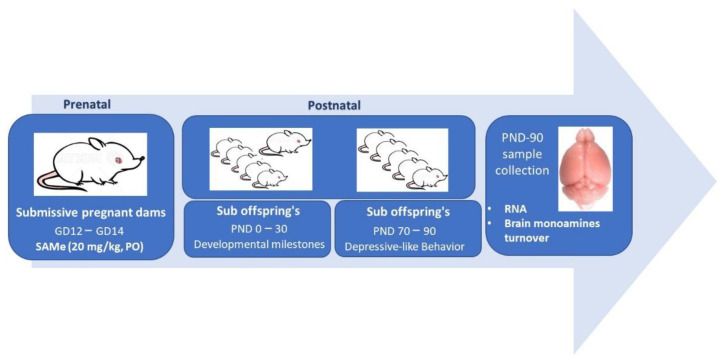
Chart flow describing experimental steps.

**Treatment:** S-adenosylmethionine (SAMe) was kindly a gift by Prof. Szyf M., (McGill University, **Faculty of Medicine**). During days 12–14 of gestation Submissive **females** were treated daily by oral gavage with 20 mg/Kg of SAMe or by Normal Saline (controls). Pregnant **females** were housed individually in cages in a colony room (12:12 L:D cycle with lights on 07:00–19:00 h, **23** ± 2 °C, ambient humidity) and provided with standard laboratory chow and water, ad libitum. Further, their offspring were separated at weaning (≈day 21) in groups of five per cage (see chart flow Figure 5).

**Brain monoamine levels measurements:** At the end of the behavioral evaluations on day 90, mice were killed by exposure to CO_2_ and their Prefrontal cortex (PFC) was collected, frozen in liquid nitrogen and stored at −80 °C for the study of monoamines and gene expression. We did not study the PFC of the wild type Sabra mice, as Sub offspring treated with SAMe were compared to Saline treated Sub mice. Dopamine (DA) and its metabolites, 3,4-dihydroxyphenylacetic acid (DOPAC), homovanillic acid (HVA); serotonin- a 5-hydroxytriptamine (5-HT) and its metabolite, 5-hydroxyindoleacetic acid (5-HIAA); norepinephrine (NE) and its metabolite, 3-methoxy-4-hydroxyphenylglycol (MHPG) contents were measured in the brain by using the HPLC ALEXYS neurotransmitters analyzer with column acquity UPLC BEH C18, **1.7 µm**, 1 × 100 mm (Waters) and electrochemical detector (ECD). For brain monoamines and their metabolites (5-HT, 5-HIAA, NE, MHPG, DA, DOPAC and HVA) analyses, the frozen PFC was prepared according to the ALEXYS™ neurotransmitter analyzer protocol.

Briefly, the tissue samples were homogenized in 0.2 M perchloric acid (0.5 mL/100 mg wet tissue), left in ice for 30 min and centrifuged at 20,000 G for 15 min at +4 °C. The supernatants were filtered (PhenexTM Nylon, 0.45 μm) and adjusted to pH 3.0 with 1 M sodium acetate.

For analysis, 2 μL of each sample was injected into the HPLC-ECD system at temperature 37 °C with flow rate 50 µL/min and backpressure about 250 bar. The mobile phase included 100 mM phosphoric acid, 100 mM citric acid, 0.1 mM EDTA-Na_2_, 6,9 mL 50% NaOH, 600 mg/L octanesulfonic acid sodium salt, 8% acetonitrile.

The quantity of monoamines in a total volume of 20 μL in each sample was measured by chromatography software of DataApex (Prague, The Czech Republic) and final concentrations expressed as ng/g of wet brain tissue were adjusted to commercially obtained standards: 5-hydroxytryptamine (5-HT, Supelco, CAS #: 50-67-9), 5- 5-hydroxyindoleacetic acid (5-HIAA, Merck, Jerusalem, Israel; CAS #: 54-16-0), dopamine (DA, Merck, Jerusalem, Israel; CAS #: 62-31-7), 3,4-Dihydroxyphenylacetic acid (DOPAC, Merck, Jerusalem, Israel; CAS #: 102-32-9), norepinephrine (NE, Merck, Jerusalem, Israel; CAS #: 51-41-2), 4-hydroxy-3-Methoxyphenylglycol (MHPG) piperazine (Merck, Jerusalem, Israel; CAS #: 67423-45-4), homovanillic acid (HVA, Supelco, Merck, Jerusalem, Israel; CAS #: 306-08-1). For 5-HT and 5-HIAA, stock standard solutions were prepared in 0.1 M acetic acid including 1 mg/mL EDTA-2Na. All other standards were prepared in 0.1 N HCl including 1 mg/mL EDTA-2Na. Standard solutions were serially diluted in 0.02 M acetic acid including 10 μM EDTA-2Na to prepare working concentrations of 250 to 0.39 pg/μL required for the calibration.

We measured the levels in the PFC of 5-HT, 5-HIAA and 5-HT turnover (5-HIAA/5-HT) and used 5-HIAA as an indicator of MAO-A activity [53]. The ratio of DOPAC to dopamine indicates the rate of dopamine metabolism, whereas changes in the levels of dopamine metabolites, DOPAC and HVA, reflect changes in MAO activity. Dopaminergic neuronal activity can be further estimated by calculation of DOPAC + HVA/dopamine ratio. This ratio, which indicates alterations in the rate of dopamine turnover, was found to be changed in a number of pathological conditions (experimental model of Parkinson’s disease or following stroke) as well as during drug treatments [54,55].

**Gene expression studies:** The, PFC was inserted in to 300 µL of DNA/RNA Shield (#Cat: ZR-R1100-50, Zymo Research, Irvine, CA, USA), frozen by liquid nitrogen and stored at −80 °C.

We studied in the PFC by real time RT-PCR the expression of genes related to serotonin synthesis *Tph2*, degradation: *Mao-a* and *Mao-b*, and serotonin receptor *Htr1a* and *Htr2a*. In addition, we studied the expression of genes related to dopamine enzymatic degradation: *Mao-a* and *Mao-b*, *Comt* and two dopamine receptors gens: D_1_R and *D_2_R* genes. The analysis was normalized to the housekeeping gene *Gapdh*. mRNAs were purified with Direct-zol™ RNA MiniPrepPlus (Cat #: ZR-R2081, Zymo Research, Irvine, CA, USA) and quantified according to absorbance at **260 nm** using spectrophotometry. Complementary DNA (cDNA) was transcribed from 1 mg RNA using qScript cDNA Synthesis Kit (Cat #:95047-100-2, Quantabio, Beverly, MA, USA). cDNA samples were analyzed for the expression of the genes using commercially synthesized primers (Merck, Israel, presented in Table 2). Amplification was performed using PerfeCTa SYBR Green FastMix (Cat #: 95074-012; Quantabio, Beverly, MA, USA) using the following conditions: 30 s denaturation at 95 °C, followed by 30 s annealing at 60 °C for a total of 40 cycles in Agilent AriaMx qPCR thermal cycler. The obtained cycle thresholds (Ct) values for gene of interest (a target sample) and housekeeping gene (GAPDH) for both the treated and untreated samples were used to calculate the relative fold gene expression level 2^−∆∆CT^ [56]. Gene symbols are written in italicized letters with only the first letter in upper-case. Protein symbols are written in regular, upper-case letters.

Statistical analysis: for statistical significance of monoamines content in brain tissue in relation to gender, we performed analysis of way variance (two-way ANOVA), followed by post hoc Bonferroni test or multiple paired *t*-test comparison analysis [57]. For all analyses, *p* ≤ 0.05 was considered significant. GraphPad Prism 9.0 software was used for Graph’s preparation and statistical analysis.

## 5. Conclusions

In our study, SAMe treatment raised the levels of several monoamines and altered the expression of genes involved in their metabolism. The “correction” of the metabolism of the monoamines in the PFC may explain our previous findings that prenatal SAMe treatment alleviated several major depressive symptoms in the Sub mice [21]. This points to the possibility that the beneficial effects of SAMe in the treatment of depression in our mouse model, and possibly in humans too, results from epigenetic modulations of gene expression. We are aware that we did not study DNA methylation, but the fact that the changes were observed after a short period of prenatal treatment and more than 3 months after treatment demonstrate the long-term changes in gene expression induced by SAMe. It is worth mentioning that in our previous studies [58] we also found that similar prenatal exposure of ICR mice to SAMe induced gender related changes in the expression of many neuropathology, inflammatory and neurogenesis genes in the PFC of newborn mice. Thus, SAMe seems to be a significant epigenetic modulator that might be of help in the alleviation of a variety of neuropsychiatric diseases of epigenetic etiology.

## Figures and Tables

**Table 1:** Summary of the monoamine levels and gene expression studies.

MONOAMINES and METABOLITES	Male	Female	GENES	Male	Female
5-HT	no change	increase ↑	*Tph2*	no change	no change
5-HIAA	no change	no change	*Mao-a*	significantly downregulated ↓	trend to decrease
DA	increases ↑	increases ↑	*Htr2a*	significantly downregulated ↓	no change
DOPAC	increase ↑	increase ↑	*Sert*	no change	no change
HVA	no change	increase ↑	*Mao-b*	significantly downregulated ↓	trend to decrease
NE	no changes	no change	*Comt*	significantly downregulated ↓	significantly downregulated ↓
MHPG	no changes	no change	*D_1_R*	significantly downregulated ↓	no change
			*D_2_R*	trend to decrease	no change

**Table 2:** List of primers used in RT-PCR.

Oligo Name	Sequence 5′ to 3′	
Glyceraldehyde 3-phosphate dehydrogenase–GAPDH	F	GGGGCTCTCTGCTCCTCCCTGT
	R	TGACCCTTTTGGCCCCACCCT
Serotonin receptor–*Htr2a*	F	CCTGATGTCACTTGCCATAGCTG
	R	CAGGTAAATCCAGACGGCACAG
Tryptophan hydroxylase 2–*Tph2*	F	GTTGATGCTGCGGCTCAGATCT
	R	GAAGCTCGTCATGCAGTTCACC
Serotonin Transporter–*Sert*	F	TCGCCTCCTACTACAACACC
	R	ATGTTGTCCTGGGCGAAGTA
Catechol-O-Methyltransferase–*Comt*	F	ACGAGGGGATGAGAGAGTCCT
	R	AGCAGCCAACAGCATTTATGGG
Monoamine oxidase A–*Mao-a*	F	CGTGATCGGAGGTGGCATTTC
	R	AAAGGCGCCCCGAAAGG-3
Monoamine oxidase B–*Mao-b*	F	GGGGGCGGCATCTCAGGTAT
	R	TGCTTCCAGAACCACCACACT
Dopamine receptor D1–D_1_R	F	AGATGACTCCGAAGGCAGCCTT
	R	GCCATGTAGGTTTTGCCTTGTGC
Dopamine receptor D2–*D_2_R*	F	TTCCCAGTGAACAGGCGGAGA
	R	TTTGGCAGGACTGTCAGGGTT

## Data Availability

Not applicable.

## Abbreviations

5-HIAA	5-hydroxyindoleacetic acid
5-HT	5-hydroxytryptamine or serotonin
HT1A	Gene 5-hydroxytryptamine receptor 1A gene
5-HT_1A_R	5-hydroxytryptamine receptor 1A
HT2A	Gene for 5-hydroxytryptamine receptor 2A
5-HT_2A_R	5-hydroxytryptamine receptor 2A
COMT	Catechol-O-methyl transferase
CSF	Cerebrospinal fluid
DA	Dopamine
DOPAC	3,4-dihydroxyphenylacetic acid
D_1_R	Dopamine receptors D_1_
D_2_R	Dopamine receptors D_2_
EPM	Elevated Plus Maze
HPC	Hippocampus
HVA	Homovanillic acid
MAO-A	Monoamine oxidase A
MAO-B	Monoamine oxidase B
MDD	Major Depressive Disorder
MHPG	4-hydroxy-3-Methoxyphenylglycol
NE	Norepinephrine
OF	Open field
PFC	Prefrontal cortex
SAMe	S-Adenosyl-methionine
SERT	Serotonin transporter
Sub mice	Submissive mice
Tph2	Tryptophan hydroxylase enzyme 2
